# Dataset on enrichment of selected trace metals in the soil from designated abandoned historical gold mine solid waste dump sites near residential areas, Witwatersrand Basin, South Africa

**DOI:** 10.1016/j.dib.2022.107895

**Published:** 2022-02-02

**Authors:** Lowanika V. Tibane, David Mamba

**Affiliations:** a Discipline of Geological Sciences, School of Agricultural, Earth and Environmental Sciences, University of KwaZulu-Natal, Westville Campus, Durban, KwaZulu Natal, South Africa; b 30 Birch Street, Lindhaven, Roodepoort, 1724, South Africa

**Keywords:** Trace elements enrichment, Geoaccumulation index, Contamination factor, Potential health risks, Land use type

## Abstract

Soil is a naturally occurring non-renewable resource, and good soil quality is a prerequisite for the survival of plants, animals and humans. Soil quality depends on the content and distribution of trace elements. Gold mining in the Witwatersrand Basin in South Africa left behind enormous unrehabilitated mining waste tailings near the City of Johannesburg that are contaminated by trace elements. Heavy metals change the physical and chemical properties of the soil derived from the mining waste material, consequently disturbing the normal functions of the soil and posing a potential health risk to plants, animals, and humans. To assess soil quality from abandoned historical gold mine residues, three conglomerate samples were petrologically examined using hand specimen, polished thin sections viewed under a reflected light microscope, and X-ray diffraction, to determine the mineralogical composition. The minerals include quartz, mica, chlorite, calcite and sulphides. Geochemical data of major elements measured by weight percent (wt%) were generated using X-ray fluorescence (XRF) technique and are NiO < Cr_2_O_3_ < V_2_O_5_ < ZrO_2_ < MnO < P_2_O_5_ < TiO_2_ < Al_2_O_3_ < CaO < MgO < Na_2_O < K_2_O < Fe_2_O_3_ < SiO_2_. The geochemistry of trace elements, pH, and electrical conductivity (EC), were determined from 21 soil samples. The samples were collected from 30 to 150 cm depths from nine sites and investigated using inductively coupled plasma optical emission spectroscopy (ICP-EOS) and XRF. ICP-EOS data (mg/kg): Fe > S > Cu > Mn > Cr > Zn > Ni > Co > Mo > P*,* and XRF data (mg/kg): *S* > F > Cl > V > Cr > Zn > Co > Cu > Ni > Mo. The trace elements data are variable in the tailing soil than in the crustal background soil. According to ICP-EOS data, the surveyed sites show increased Cu content, pH values of 1.9–5.3 and EC values of 43–679 mS/m, indicating soil contamination.

## Specifications Table

**Table:** 

Subject	Environmental science
Specific subject area	Geochemistry and petrology, environmental chemistry, ecological risk
Type of data	Tables, images, charts, graphs, and figures
How the data were acquired	Trenches were dug to the depth of 30 cm to 150 cm using a spade (Fig. 2). Two soil samples were collected from each mine tailing dump site, where the first sample was taken from upstream and second sample was taken from downstream (Fig. 1). A minimum of 500 g of soil was collected from each location and placed inside brown amber glass bottles to avoid reaction with sun rays. The bottles were placed inside a cool box at the temperature below 25 °C and transported to the laboratory with 24 h for trace elements analyses using inductively coupled plasma optical emission spectrometry (ICP-OES) (Section 2.2.4). Three conglomerate samples were collected from Mogale City, New Canada and Kagiso and investigated utilising polished thin sections viewed under a reflected light microscope (Section 2.2.1), X-ray diffraction (XRD) (Section 2.2.2), and X-ray fluorescence (XRF) (Section 2.2.3).
Data format	Raw, analysed, processed, and filtered data [1]
Description of data collection	Images of rocks (Fig. 2A and B) and soil samples (Fig. 3) were taken with a digital camera. Photomicrographs were taken as JPEGs under the microscope (Fig. 2C and D), mineralogy was determined using thin sections and confirmed by the XRD data provided in the Excel file and shown in Table 2. Soil samples are medium-grained (0.5–2 mm in diameter) and range from grey to brown to reddish to yellow. XRF data in Excel format describes soil chemistry given as oxides in weight percent (wt%), and ICP-EOS data describe the soil pH value, electrical conductivity (mS/m), and trace element data (mg/kg), normalised by using STDDS10 and STDOXC109 reference standards in combination with a background blank value for instrument calibration. To illustrate the quality of the soil samples investigated, the contamination factor (Table 5), degree of contamination (Table 7), and geoaccumulation index (Table 8) were compared to the background concentration of the selected metals.
Data source location	The sampling locations are shown in Fig. 1 and the exact coordinates were recorded using mobile smart phones equipped with Global Positioning System (GPS) application and provided in Table 1.
Data accessibility	Repository name: Mendeley Data Data identification number: doi: 10.17632/wxw27gz8rj.1 Direct link to the dataset: https://data.mendeley.com/datasets/wxw27gz8rj/1

## Value of the Data

• The data provide information on the level of soil contamination, which causes the contamination of the streams in the area and polluting the air with dust from the tailings.
• The residents of the investigated sites and all interested parties, can learn about the potential health risks associated with unbalanced concentration of trace elements in the soil derived from gold mine dumps.
• Similar local scientific research or studies from other parts of the world experiencing similar contamination challenges, can adopt and apply the methods that were utilised in this work for planning, designing, developing, and implanting soil contamination control strategies.

## 1 Data Description

**Fig. 1:**
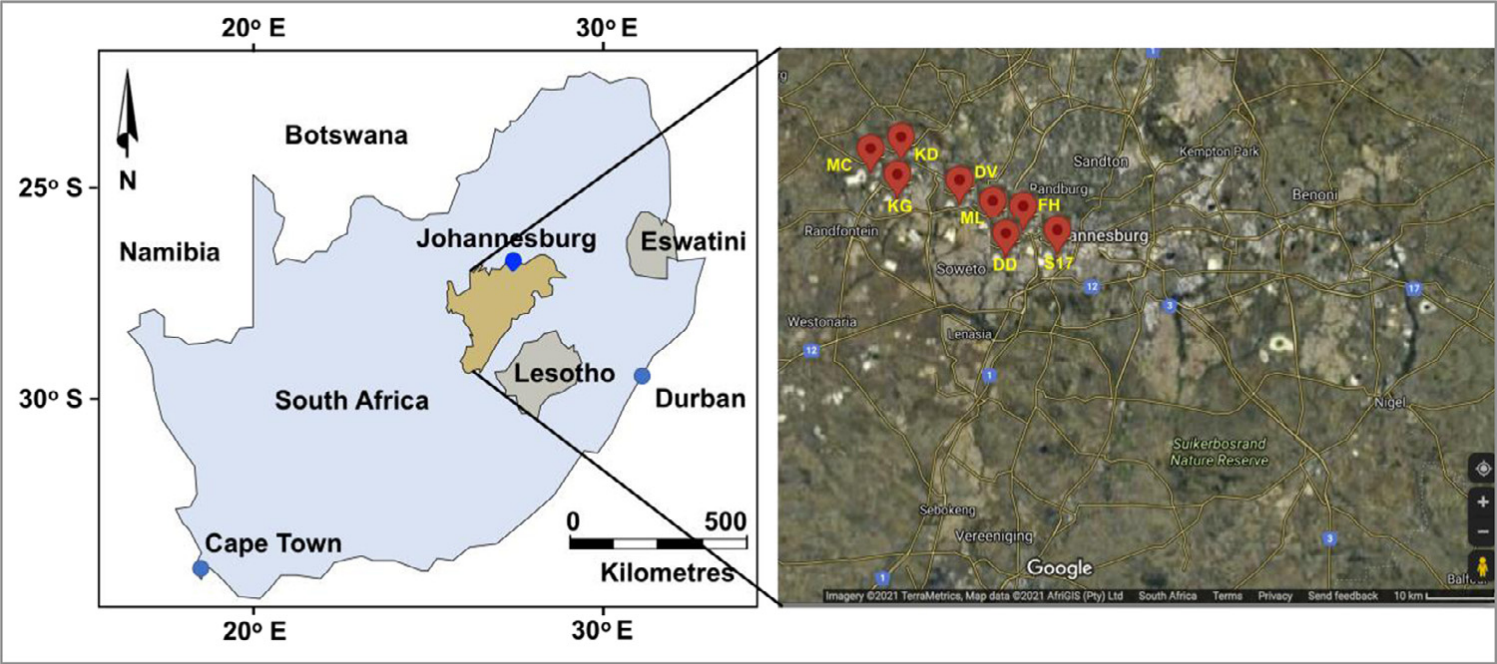
A map of South Africa indicating the location of the Witwatersrand Basin on the left and the sampling locations on the right, redrawn after [2], using CorelDRAW Graphics Suite 2021. MC = Mogale City, KG = Kagiso, KD = Krugersdorp, DV = Davidsonville, ML = Marie Lewis, FH = Fleurhof, DD = Durban Deep, and S17 = Shaft 17.

South Africa is a host to a large gold deposit in the Witwatersrand Basin and has been mining gold around the City of Johannesburg since 1886 [2,3]. During the operation of the mines, a large number of mine dumps were created (Fig. 1), and most of them were abandoned without rehabilitation at the end of the mining work. The chemical compsition of the tailings includes sulphides, mainly pyrrhotite, pyrite, chalcopyrite (Table 2) and elevated content of trace elements [4]. Increased sulphide content can lead to acid mine drainage [3], subsequently contaminating the surface and underground water and soil. The collected data show that the quality of soil is affected by the unbalanced concentration levels of the trace elements such as Cr, Co, Fe, Mn, Mo, Ni, Co, P, Zn, and S. Soil contamination poses potential health risks to the people living near tailings.

**Fig. 2:**
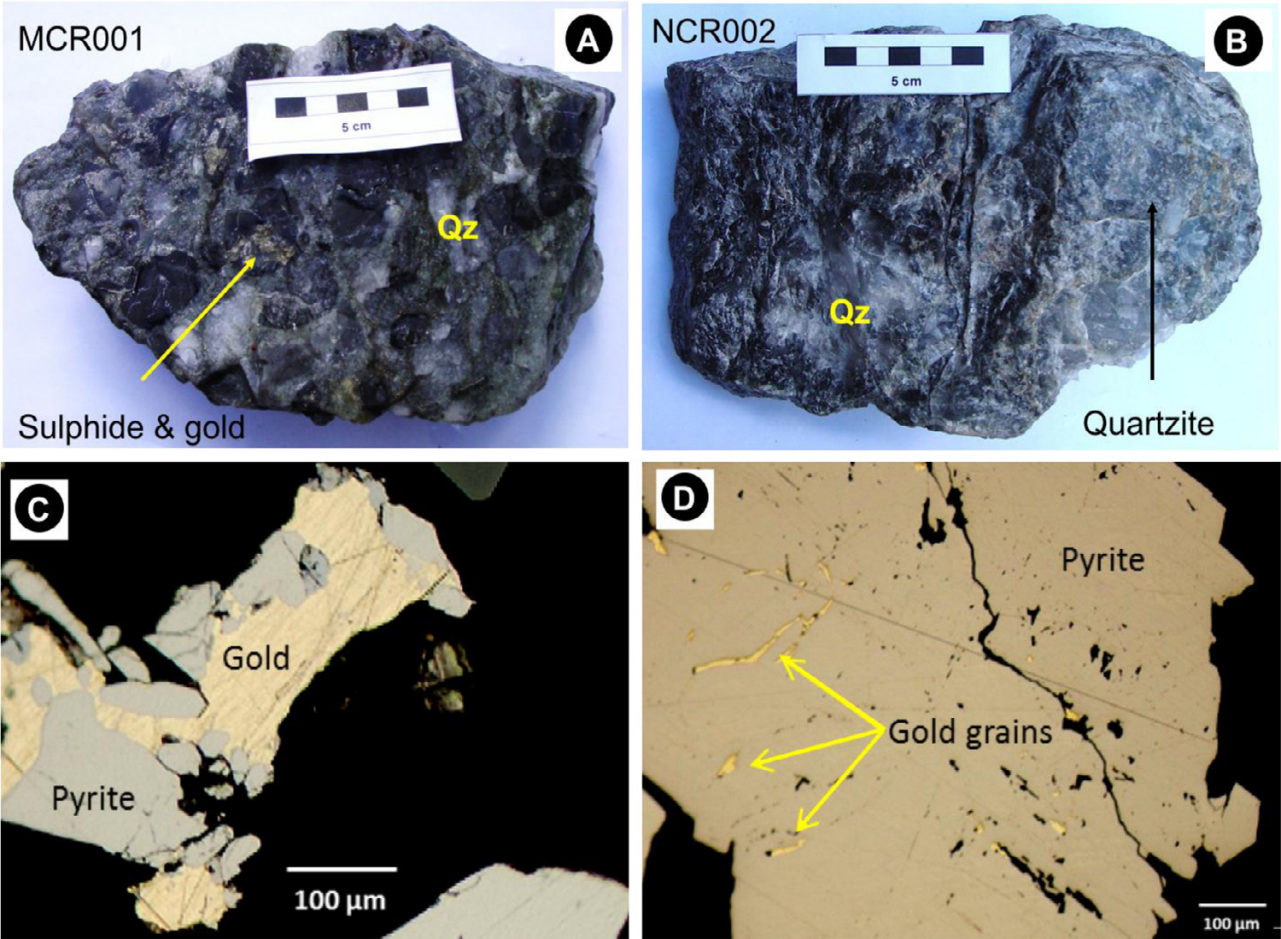
(A) – conglomerate from Mogale City (MCR001) and (B) – New Canada (NCR002). Both samples are consisting of quartz pebbles (Qz) and quartzite clasts in the matrix composed of sulphides (e.g. pyrite), muscovite, and clay minerals. Pyrite grains are brownish, anhedral, averaging to ca. 400 µm in diameter, and contain inclusions of gold (C and D). Gold is yellowish, subhedral to elongated and 40–300 µm and embedded within pyrite grains.

**Fig. 3:**
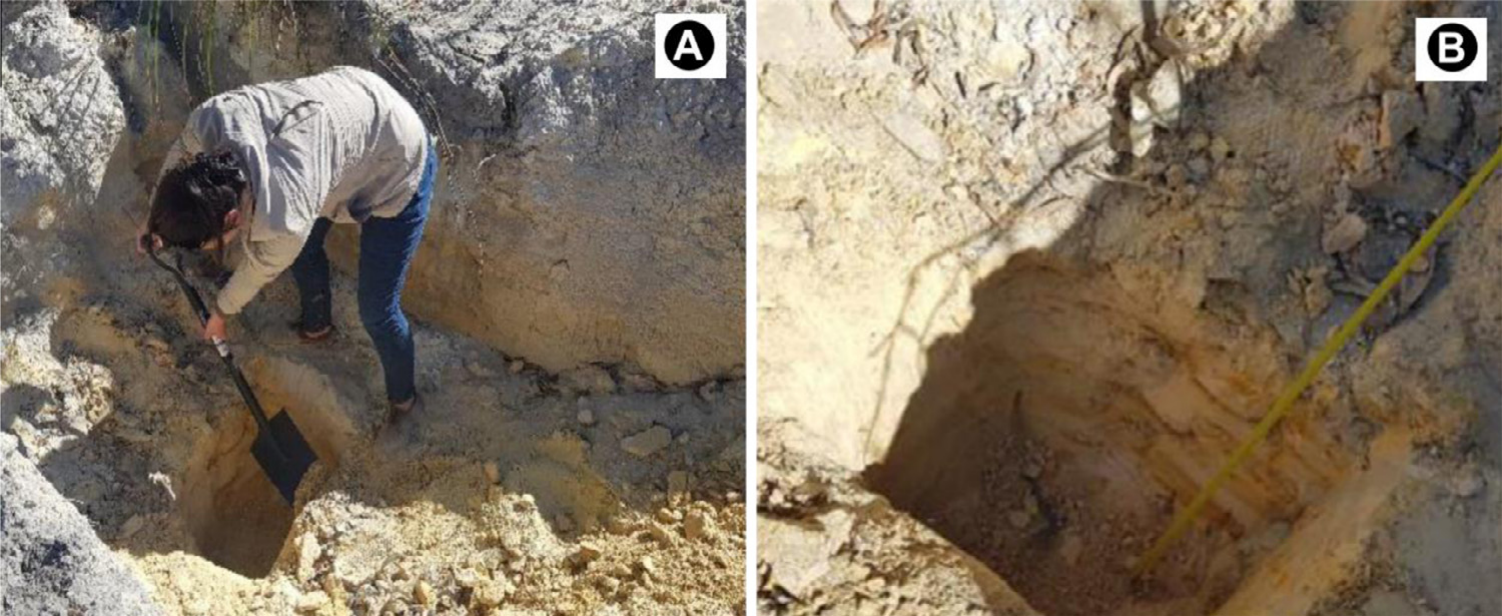
(A) – trenching at Durban Deep using a spade, (B) – soil identification, description and sampling (Table 1)*.*

**Table 1:** Sampling locations and sample description.

Location	Farm Name	Coordinates	Elevation (m)	Sample ID and Description
Krugersdorp	Luipaardsvlei 246 IQ	S26°06′32″; E27°45′55″	1708	KDS1 is yellow in colour and contains whitish crystals of gypsum deposited on the surface. Dark-brown rootlets are present in KDS2. These samples are coarse-grained and comprise sand and stone fragments.
Kagiso	Luipaardsvlei 246 IQ	S26°08′13″; E27°46′06″	1675	KGS1 is brown, coarse-grained ca. 1 to 2.5 mm in diameter and contains organic matter. KGS2 and KGS3 are grayish, fine-grained and ca. 1 mm in diameter.
Mogale City	Waterval 174 IQ, Krugersdorp, 1739	S26°07′13″; E27°43′15″	1667	MCS1 is yellow, covered with gypsum crystals. Dark-brown plant rootlets occur in MCS2. These samples are composed mainly of sandy grains intermingled with white stones.
Durban Deep	Roodepoort 237-Iq, near Langlaagte Farm	S26°10′50′'; E27°52′34″	1676	DDS1 and DDS2 are reddish and soaked in reddish water ponds (Fig. 3) and covered with whitish powdery salt deposits.
Marie Lewis	M77, Meadowlands, Roodepoort, 1724	S26°11′07″; E27°52′51″	1670	Sample MLS1 and MLS2 are Hutton type, reddish-brown to red, consisting of an orthic top layer [3], and characterised with clay to sandy particles.
Davidsonville	Roodepoort, 1724	S26°09′29″; E27°51′03″	1690	DVS1 is a coarse-grained and reddish-brown. DVS2 and DVS3 are fine-grained and contain dark organic materials.
Fleurhof	Helpmekaar Rd, Vogelstruisfontein, Randburg, 1709	S26°11′39″; E27°54′07″	1670	FHS1, FHS2, and FHS3 are coarse-grained with particles greater than 2 mm in diameter. Their brown colour is the result of organic components.
Shaft 17	Mooifontein 225-Iq, Johannesburg, 2093	S26°13′36″; E27°58′38″	1680	Coarse-grained particles of 1 mm diameter occur in yellow S17S1 and S17S2. These samples are devoid of organic components.
New Canada	Mooifontein 225-Iq, Johannesburg, 2093	S26°12′42″; E27°57′13″	1685	NCS1, NCS2 are fine-grained, but contain gravel size stones of 5 mm in diameter. Organic materials occur and give these samples a blackish colour.

**Table 2:** Raw XRD data reported in weight percentage (wt%) for mineralogical composition of the conglomerate samples [1].

Sample ID	Calcite	Chalcopyrite	Chlorite	Muscovite	Pyrite	Pyrrhotite	Quartz	Total
MCR001	2.55	0.16	18.50	24.56	0.12	3.13	50.98	100.00
NCR002	2.19	0.30	18.92	23.90	0.24	2.96	51.29	99.80
KGR003	2.41	0.19	18.12	25.02	0.19	3.27	51.52	100.72
Average	2.38	0.22	18.51	24.49	0.18	3.12	51.26	100.17

Table 1 contains location name, farm name, coordinates, altitude, sample identification, and sample description for a total of 21 soil samples including three duplicates, which are KGS3, DVS3, and FHS3. Three samples of conglomerate lithology have been collected from three separate locations. Fig. 2A shows a sample of a conglomerate rock from Mogale City (MCR001) located at Waterval 174 IQ farm in Krugersdorp 1739 at (26°07′13′′S; 27°43′15′′E), and (B) shows NCR002 from New Canada in Mooifontein 225-Iq farm, Johannesburg, 2093 at (26°12′42″S; 27°57′13″E). Sample KGR003 was collected from Kagisoat (26°06′32″S; 27°45′55″E) in Luipaardsvlei 246 IQ. The conglomerate hand samples consist of 50% quartz pebbles (Qz) and quartzite clasts combined in a matrix composed of ca. 5% sulphides, 20% muscovite, and 25% clay minerals visible to the naked eye (Fig. 2A and B), supported by thin section data (Fig. 2C and D) as well as the XRD data (Table 2).

The quartz pebbles are grey, generally rounded with an average grain size of 2.5 cm in diameter as in sample MCR001 (Fig. 2A and B). The quartzite clasts are ranging from 3.0 – 5 cm in diameter, sub-angular to sub-rounded, grey in colour such as in NCR002 (Fig. 2B). Muscovite occurs as fine grains of thin brown flakes in mm scale in the matrix, together with fine greenish grains of chlorite, and yellowish subhedral to euhedral, 0.5 – 1.0 mm diameter sulphides which are mainly pyrrhotite, pyrite and chalcopyrite (Fig. 2C and D). Gold occurs in association with the sulphides (Fig. 2C) and as inclusions in pyrite that are occupying cracks as veinlets (Fig. 2C). According to the XRD data (Table 2), calcite contributes an average of 2.38 wt% [1] and according to polished thin section petrograph, calcite occurs and forms the cement that is binding the clasts and the matrix. The general chemical formula for the minerals are pyrite (FeS_2_), chalcopyrite (CuFeS_2_), calcite (CaCO_3_), pyrrhotite (Fe_1-x_S), quartz (SiO_2_), chlorite [(Mg,Fe)_3_(Si,Al)_4_O_10_(OH)_2_•(Mg,Fe)_3_(OH)_6_)], muscovite (KAl_2_(AlSi_3_O_10_)(F,OH)_2_.

X-ray fluorescence (XRF) data (Table 3) for major elements show NiO < Cr_2_O_3_ < V_2_O_5_ < ZrO_2_ < MnO < P_2_O_5_ < TiO_2_ < Al_2_O_3_ < CaO < MgO < Na_2_O < K_2_O < Fe_2_O_3_ < SiO_2_, measured in weight percentage (wt%). Moreover, the XRF techniques was performed for trace elements analyses (Table 4) and the data show *S* > F > Cl > V > Cr > Zn > Co > Cu > Ni > Mo, recorded in (mg/kg). The XRF data for trace elements are comparable to the ICP-OES data in Table 6.

**Table 3:** Raw XRF data reported in weight percentage (wt%) for the conglomerate major elements, loss on ignition (L.O.I) at 1100 °C, all Fe is reported as Fe_2_O_3_. Stdev = standard deviation, the dash (–) = not calculated [1].

Sample ID	SiO_2_	TiO_2_	Al_2_O_3_	Fe_2_O_3_	MnO	MgO	CaO	Na_2_O	K_2_O	P_2_O_5_	Cr_2_O_3_	NiO	V_2_O_5_	ZrO_2_	LOI	Total
**MCR001**	65.80	0.68	14.67	3.75	0.06	2.30	2.50	3.77	4.63	0.28	0.01	<0.01	0.01	0.03	1.32	99.82
**NCR002**	65.56	0.67	15.27	3.72	0.05	2.32	2.68	3.80	4.60	0.28	0.01	0.02	0.01	0.02	1.29	100.31
**KGR003**	68.44	0.91	10.67	9.36	0.14	2.70	1.82	0.09	2.56	0.12	0.02	0.01	0.02	0.02	3.41	100.29
**Average**	66.60	0.75	13.54	5.61	0.08	2.44	2.33	2.55	3.93	0.23	0.02	0.01	0.02	0.02	2.01	100.14
**Stdev**	0.40	0.03	0.30	0.30	0.01	0.10	0.07	0.11	0.06	0.08	0.01	0.01	<0.01	0.01	<0.01	–

**Table 4:** Raw XRF data (mg/kg) for trace elements of the three conglomerate samples, Stdev = standard deviation [1].

	Essential trace elements (mg/kg)									
Sample ID	S	F	Cl	V	Cr	Zn	Co	Cu	Ni	Mo
**MCR001**	140.00	1,050.00	450.00	65.00	55.00	48.00	65.00	20.00	34.00	1.20
**NCR002**	455.00	2,249.00	707.00	55.00	47.00	50.00	69.00	24.00	37.00	1.00
**KGR003**	7,120.00	484.00	34.00	132.00	126.00	113.00	64.00	125.00	74.00	5.00
**Average**	2,572.00	1,261.00	397.00	84.00	76.00	70.00	66.00	56.00	48.00	3.00
**Stdev**	56.00	2.00	48.00	70.05	396.73	65.88	75.90	1,261.05	83.94	2,571.76

Table 5 provides the raw data of selected trace elements such as P, Co, Cr (t), Cu, Fe, Mn, Mo, Ni, S, Zn, as well as the pH value and electrical conductivity of the soil acquired by using ICP-EOS method (Section 2.2.4). Table 5 includes statistical data, such as minimum, maximum, average, and standard deviation for each research location. The crustal metal concentration and a contamination factor are given at the end of Table 5, used to determine geoaccumulation index (Table 8) and the levels of soil contamination (Table 7). The duplicate samples (KGS3, DVS3, and FHS3) data corelate well with their corresponding sample analyses KGS2, DVS2, and FHS2 respectively from the same locations. Therefore, the duplicates data were excluded in the calculations of the statistical data in Table 5, but were considered for judging the accuracy of the raw data, available in Mendeley Data and accessible via a link provided in specification table.

**Table 5:** Raw ICP-OES data for the concentration of trace elements (mg/kg), electrical conductivity (EC) in mS/m, pH, and statistical data for each sampling location. The dashes (–) = not determined.

		Electrical										
Variables	pH	conductivity	P	Co	Cr(t)	Cu	Fe	Mn	Mo	Ni	S	Zn
**Kagiso**												
KGS1	3.10	351.00	0.32	0.50	162.00	50.00	81,400.00	1,630.00	0.50	28.00	7,020.00	77.00
KGS2	5.30	43.00	0.45	0.50	120.00	167.00	40,100.00	292.00	1.60	136.00	1,170.00	108.00
Average	4.20	197.00	0.39	0.50	141.00	108.50	60,750.00	961.00	1.05	82.00	4,095.00	92.50
STDEV	1.56	217.79	0.09	0.00	29.70	82.73	29,203.51	946.11	0.78	76.37	4,136.57	21.92
Min	3.10	43.00	0.32	0.50	120.00	50.00	40,100.00	292.00	0.50	28.00	1,170.00	77.00
Max	5.30	351.00	0.45	0.50	162.00	167.00	81,400.00	1,630.00	1.60	136.00	7,020.00	108.00

**Krugersdorp**												
KDS1	2.90	303.00	0.26	0.50	52.00	41.00	36,800.00	143.00	0.50	29.00	5,600.00	42.00
KDS2	2.80	175.00	0.26	0.50	79.00	119.00	30,500.00	139.00	2.40	33.00	4,240.00	71.00
Average	2.85	239.00	0.26	0.50	65.50	80.00	33,650.00	141.00	1.45	31.00	4,920.00	56.50
STDEV	0.07	90.51	0.00	0.00	19.09	55.15	4,454.77	2.83	1.34	2.83	961.67	20.51
Min	2.80	175.00	0.26	0.50	52.00	41.00	30,500.00	139.00	0.50	29.00	4,240.00	42.00
Max	2.90	303.00	0.26	0.50	79.00	119.00	36,800.00	143.00	2.40	33.00	5,600.00	71.00

**Mogale City**												
MCS1	4.00	182.00	0.24	0.50	111.00	225.00	8,640.00	183.00	0.50	83.00	2,310.00	66.00
MCS2	4.90	217.00	0.21	0.50	74.00	24.00	31,700.00	7,450.00	0.50	22.00	1,460.00	34.00
Average	4.45	199.50	0.23	0.50	92.50	124.50	20,170.00	3,816.50	0.50	52.50	1,885.00	50.00
STDEV	0.64	24.75	0.02	0.00	26.16	142.13	16,305.88	5,138.54	0.00	43.13	601.04	22.63
Min	4.00	182.00	0.21	0.50	74.00	24.00	8,640.00	183.00	0.50	22.00	1,460.00	34.00
Max	4.90	217.00	0.24	0.50	111.00	225.00	31,700.00	7,450.00	0.50	83.00	2,310.00	66.00

**Davidsonville**												
DVS1	3.40	71.00	0.24	0.50	246.00	26.00	42,900.00	94.00	0.50	19.00	1,670.00	12.00
DVS2	4.40	258.00	0.22	0.50	134.00	26.00	27,600.00	214.00	0.50	52.00	5,960.00	82.00
Average	3.90	164.50	0.23	0.50	190.00	26.00	35,250.00	154.00	0.50	35.50	3,815.00	47.00
STDEV	0.71	132.23	0.01	0.00	79.20	0.00	10,818.73	84.85	0.00	23.33	3,033.49	49.50
Min	3.40	71.00	0.22	0.50	134.00	26.00	27,600.00	94.00	0.50	19.00	1,670.00	12.00
Max	4.40	258.00	0.24	0.50	246.00	26.00	42,900.00	214.00	0.50	52.00	5,960.00	82.00

**Fleurhof**												
FHS1	4.80	263.00	12.00	0.50	66.00	59.00	55,500.00	254.00	0.70	33.00	5,050.00	82.00
FHS2	3.50	102.00	24.00	0.50	3.20	13.00	759.00	15.00	0.50	2.50	341.00	19.00
Average	4.15	182.50	18.00	0.50	34.60	36.00	28,129.50	134.50	0.60	17.75	2,695.50	50.50
STDEV	0.92	113.84	8.49	0.00	44.41	32.53	38,707.73	169.00	0.14	21.57	3,329.77	44.55
Min	3.50	102.00	12.00	0.50	3.20	13.00	759.00	15.00	0.50	2.50	341.00	19.00
Max	4.80	263.00	24.00	0.50	66.00	59.00	55,500.00	254.00	0.70	33.00	5,050.00	82.00

**New Canada**												
NCS1	5.20	111.00	23.00	24.00	46.00	102.00	26,900.00	113.00	0.70	98.00	5,730.00	253.00
NCS2	5.20	113.00	0.20	0.50	55.00	103.00	19,900.00	77.00	0.80	53.00	1,370.00	90.00
Average	5.20	112.00	11.60	12.25	50.50	102.50	23,400.00	95.00	0.75	75.50	3,550.00	171.50
STDEV	0.00	1.41	16.12	16.62	6.36	0.71	4,949.75	25.46	0.07	31.82	3,082.99	115.26
Min	5.20	111.00	0.20	0.50	46.00	102.00	19,900.00	77.00	0.70	53.00	1,370.00	90.00
Max	5.20	113.00	23.00	24.00	55.00	103.00	26,900.00	113.00	0.80	98.00	5,730.00	253.00

**Shaft 17**												
S17S1	4.60	226.00	0.20	44.00	19.00	17.00	10,400.00	46.00	0.50	23.00	4,270.00	31.00
S17S2	5.20	50.00	0.23	0.50	67.00	104.00	20,800.00	93.00	1.60	30.00	1,100.00	116.00
Average	4.90	138.00	0.22	22.25	43.00	60.50	15,600.00	69.50	1.05	26.50	2,685.00	73.50
STDEV	0.42	124.45	0.02	30.76	33.94	61.52	7,353.91	33.23	0.78	4.95	2,241.53	60.10
Min	4.60	50.00	0.20	0.50	19.00	17.00	10,400.00	46.00	0.50	23.00	1,100.00	31.00
Max	5.20	226.00	0.23	44.00	67.00	104.00	20,800.00	93.00	1.60	30.00	4,270.00	116.00

**Marie Lewis**												
MLS1	1.90	679.00	0.25	0.50	50.00	111.00	20,9000.00	254.00	1.90	18.00	26,700.00	132.00
MLS2	2.50	424.00	0.20	7.90	2.10	24.00	12,800.00	32.00	0.60	21.00	17,300.00	20.00
Average	2.20	551.50	0.23	4.20	26.05	67.50	110,900.00	143.00	1.25	19.50	22,000.00	76.00
STDEV	0.42	180.31	0.04	5.23	33.87	61.52	138,734.35	156.98	0.92	2.12	6,646.80	79.20
Min	1.90	424.00	0.20	0.50	2.10	24.00	12,800.00	32.00	0.60	18.00	17,300.00	20.00
Max	2.50	679.00	0.25	7.90	50.00	111.00	209,000.00	254.00	1.90	21.00	26,700.00	132.00

**Durban Deep**												
DDS1	3.40	106.00	0.30	29.00	17.00	20,000.00	56.00	1.10	7.00	38.00	1.00	0.25
DDS2	2.90	339.00	0.20	62.00	38.00	9,700.00	46.00	0.50	28.00	9.50	1.00	0.86
Average	3.15	222.50	0.25	45.50	27.50	14,850.00	51.00	0.80	17.50	23.75	1.00	0.56
STDEV	0.35	164.76	0.07	23.33	14.85	7,283.20	7.07	0.42	14.85	20.15	0.00	0.43
Min	2.90	106.00	0.20	29.00	17.00	9,700.00	46.00	0.50	7.00	9.50	1.00	0.25
Max	3.40	339.00	0.30	62.00	38.00	20,000.00	56.00	1.10	28.00	38.00	1.00	0.86

**Crustal concentration and contamination factor**												
B_n_	–	–	700.00	19.00	90.00	45.00	47,200.00	850.00	2.60	68.00	2,400.00	95.00
CF	–	–	0.12	4.69	1.69	39.89	0.77	0.81	31.03	1.74	2.15	1.54

Table 6 shows the comparison between the trace element (Mo, Co, Ni, Zn, Cr, Cu, and S) concentration obtained by the XRF method and the metal content from ICP-OES technology. Fe and Mn were not reported as trace elements in the XRF data because they were reported as oxides in the major elements’ dataset. Table 6 shows changes of Co, Cu, and S between XRF data and ICP-OES data. These differences are entirely due to the fact that the XRF data comes from conglomerate samples whose composition is given by the XRD method (Table 2), while the ICP-OES data (Table 5) comes from soil samples from tailings. The tailing samples represent the geological mixture of the mined conglomerate reefs and metal introduced during gold recovery, whilst the XRF data resemble the metal composition of the parent conglomerate from the reef.

**Table 6:** Overall average XRF concentration for three conglomerate samples and overall average ICP-OES data for 21 soil samples. The dashes (–) = not determined.

Element	Symbol	XRF	ICP-OES
Molybdenum	Mo	3.00	4.20
Cobalt	Co	66.00	30.35
Nickel	Ni	48.33	38.30
Zinc	Zn	70.33	68.71
Chromium	Cr	76.00	70.60
Manganese	Mn	–	580.60
Copper	Cu	56.33	1,627.00
Sulphur	S	2,571.67	5,071.83
Iron	Fe	–	3,4516.10

Table 7 shows the geoaccumulation index data used with Table 8 to describe contamination levels. Generally speaking, the data in Table 8 has both negative and positive values. Negative values ​​represent non-contaminated soil, and positive values ​​describe contamination.

**Table 7:** Muller's classification for the geoaccumulation index (I_geo_) [5].

I_geo_ value	Class	Quality of sediment
≤ 0	0	Uncontaminated
0 – 1	1	From uncontaminated to moderately contaminated
1 – 2	2	Moderately contaminated
2 – 3	3	From moderately to strongly contaminated
3 – 4	4	Strongly contaminated
4 – 5	5	From strongly to extremely contaminated
≥6	6	Extremely contaminated

**Table 8:** Geoaccumulation index (I_geo_) processed data of the essential metals (mg/kg) in the soil samples per site for the 9 selected mine tailings in the Witwatersrand Basin of South Africa.

Elements	Kagiso	Krugersdorp	Mogale City	Davidsonville	Fleurhof	New Canada	Shaft 17	Marie Lewis	Durban Deep	Average
P	−11.41	−11.98	−12.19	−12.16	−5.87	−6.50	−12.25	−12.19	−12.04	−10.73
Co	−5.83	−5.83	−5.83	−5.83	−5.83	−5.83	0.63	−1.85	0.67	−3.95
Mn	−0.41	−3.18	1.58	−3.05	−3.24	−3.75	−4.20	−3.16	−10.64	−3.34
Fe	−0.22	−1.07	−1.81	−1.01	−1.33	−1.60	−2.18	0.65	−10.44	−2.11
Mo	−1.89	−1.43	−2.96	−2.96	−2.70	−2.38	−1.89	−1.64	2.17	−1.74
Zn	−0.62	−1.33	−1.51	−1.60	−1.50	0.27	−0.96	−0.91	−7.37	−1.73
Ni	−0.31	−1.72	−0.96	−1.52	−2.52	−0.43	−1.94	−2.39	−2.10	−1.54
Cr(t)	0.06	−1.04	−0.55	0.49	−1.96	−1.42	−1.65	−2.37	−2.30	−1.19
S	0.19	0.45	−0.93	0.08	−0.42	−0.02	−0.42	2.61	−11.81	−1.14
Cu	0.68	0.25	0.88	−1.38	−0.91	0.60	−0.16	0.00	7.78	0.86

Table 9 contains the background values ​​of the allowable trace element concentrations for various land uses in South Africa. Soil screening values ​​(SSV) for the metal contents are given in Table 9. The allowable background SSV value was compared with the measured metal content and used as a guide to assess soil contamination. Table 10 shows the difference between the measured metal value (Table 5) and the allowable value (Table 9). In Table 10, a positive value indicates contamination, and a negative value indicates uncontaminated soil.

**Table 9:** Soil Screening Values (SSV) for metal concentration (mg/kg) and South African regional guidelines for maximum permissible metal concentrations in the soil [5,7].

Element	Water Resource Land-Uses Protective SSV1	Informal Residential SSV2	Standard Residential SSV2	Commercial Industrial SSV2
Co	300.00	300.00	630.00	5,000.00
Ni	91.00	620.00	1,200.00	10,000.00
Zn	240.00	9,200.00	19,000.00	150,000.00
Cr(VI)	6.50	6.00	13.00	40.00
Mn	740.00	740.00	1,500.00	12,000.00
Cu	16.00	1,100.00	2,300.00	19,000.00

**Table 10:** Processed data (mg/kg) for the calculation of the difference between measured metal concentration and the maximum permissible background metal concentrations in the South African soil [6,7].

Water Resource Land-Uses Protective SSV1									
Analyte Name	Kagiso	Krugersdorp	Mogale City	Davidsonville	Fleurhof	New Canada	Shaft 17	Marie Lewis	Durban Deep
**Water Resource Land-Uses Protective SSV1**									
Co	−299.50	−299.50	−299.50	−299.50	−299.50	−276.00	−256.00	−292.10	−254.50
Ni	−9.00	−60.00	−38.50	−55.50	−73.25	−15.50	−64.50	−71.50	−67.25
Zn	−147.50	−183.50	−190.00	−193.00	−189.50	−68.50	−166.50	−164.00	−239.14
Cr(t)	−45865.50	−45941.00	−45914.00	−45816.50	−45971.90	−45956.00	−45963.50	−45980.45	−45979.00
Mn	221.00	−599.00	3076.50	−586.00	−605.50	−645.00	−670.50	−597.00	−739.20
Cu	92.50	64.00	108.50	10.00	20.00	86.50	44.50	51.50	14834.00

**Informal Residential SSV2**									
Co	−299.50	−299.50	−299.50	−299.50	−299.50	−276.00	−256.00	−292.10	−254.50
Ni	−538.00	−589.00	−567.50	−584.50	−602.25	−544.50	−593.50	−600.50	−596.25
Zn	−9107.50	−9143.50	−9150.00	−9153.00	−9149.50	−9028.50	−9126.50	−9124.00	−9199.14
Cr(t)	−45865.00	−45940.50	−45913.50	−45816.00	−45971.40	−45955.50	−45963.00	−45979.95	−45978.50
Mn	221.00	−599.00	3076.50	−586.00	−605.50	−645.00	−670.50	−597.00	−739.20
Cu	−991.50	−1020.00	−975.50	−1074.00	−1064.00	−997.50	−1039.50	−1032.50	13750.00

**Standard Residential SSV2**									
Co	−629.50	−629.50	−629.50	−629.50	−629.50	−606.00	−586.00	−622.10	−584.50
Ni	−1118.00	−1169.00	−1147.50	−1164.50	−1182.25	−1124.50	−1173.50	−1180.50	−1176.25
Zn	−18907.50	−18943.50	−18950.00	−18953.00	−18949.50	−18828.50	−18926.50	−18924.00	−18999.14
Cr(t)	−95872.00	−95947.50	−95920.50	−95823.00	−95978.40	−95962.50	−95970.00	−95986.95	−95985.50
Mn	−539.00	−1359.00	2316.50	−1346.00	−1365.50	−1405.00	−1430.50	−1357.00	−1499.20
Cu	−2191.50	−2220.00	−2175.50	−2274.00	−2264.00	−2197.50	−2239.50	−2232.50	12550.00

**Commercial Industrial SSV2**									
Co	−4999.50	−4999.50	−4999.50	−4999.50	−4999.50	−4976.00	−4956.00	−4992.10	−4954.50
Ni	−9918.00	−9969.00	−9947.50	−9964.50	−9982.25	−9924.50	−9973.50	−9980.50	−9976.25
Zn	−149907.50	−149943.50	−149950.00	−149953.00	−149949.50	−149828.50	−149926.50	−149924.00	−149999.14
Cr(t)	−789899.00	−789974.50	−789947.50	−789850.00	−790005.40	−789989.50	−789997.00	−790013.95	−790012.50
Mn	−11039.00	−11859.00	−8183.50	−11846.00	−11865.50	−11905.00	−11930.50	−11857.00	−11999.20
Cu	−18891.50	−18920.00	−18875.50	−18974.00	−18964.00	−18897.50	−18939.50	−18932.50	−4150.00

## 2 Experimental Design, Materials and Methods

### 2.1 Field and sampling procedure

Mobile smartphone equipped with global positioning system was utilised to record coordinates to determine the exact sampling location (Fig. 1; Table 1). In order to investigate the concentration and contamination of the soil, 21 samples were collected from 9 abandoned mine dumps in the Witwatersrand Basin (Table 1). The sampling area extends for more than 40 km from Mogale City in the northwest of Johannesburg to Shaft No. 17 in the south (Fig. 1). The sampled tailings are 2000 m to 10 km apart. The samples were collected during winter in July 2017. No seasonal variations were considered. In general, the concentration of the trace elements in the soil tends to be minimum during summer because of excessive rain water (Naicker et al., 2003). The samples are representative of the area investigated and a minimum of two samples were collected at each tailing location near the streams draining the tailings. The first sample was collected upstream and the second sample was collected downstream. Three of the 21 samples were duplicates and were identified as KGS3, DVS3 and FHS3 from Kagiso, Davidsonville and Fleurhof respectively. These samples were analysed at separate laboratory for quality control. After removing the top 30 cm using a shovel, samples were taken at random intervals from 30 to 150 cm deep (Fig. 3). To avoid contamination from previous samples, the shovel was washed with distilled water after each sampling point. The samples were placed in glass bottle containers and transported to the laboratory in a cool box at the temperature below 25 °C within 24 h and analysed for trace element data using ICP-EOS method (Section 2.2.4). Three conglomerate samples were collected and analysed using polished thin section viewed under a reflected light microscope (Section 2.2.1), and complemented by XRD data (Section 2.2.2) and XRF data (Section 2.2.3). The graphs and tables were created using Microsoft Office Excel (2016 version).

### 2.2 Laboratory methods

#### 2.2.1 Petrography

Three conglomerate samples were collected from Mogale City (MCR001), New Canada (NCR002) and Kagiso(KGR003) (Fig. 2A and B). These samples have been described in terms of texture, size and shape and various quartz pebbles, quartzite clasts and minerals have been identified in hand specimen and under a microscope using polished thin sections. The identified minerals (Fig. 2C and D) were confirmed by XRD method (Table 2).

#### 2.2.2 XRD

The samples were prepared for XRD analysis using a back-loading preparation method. They were analysed with a PANalytical X'Pert Pro-powder diffractometer with X'Celerator detector and variable divergence- and receiving slits with Fe filtered Co-Kα radiation. The phases were identified using X'Pert Highscore plus software. The relative phase amounts in weight percentage (wt%), see Table 2, were estimated using the Rietveld method (Autoquan Program) [8]. Errors are on the 3-sigma level in the column to the right of the amount. Amorphous phases, if present, were not considered in the quantification [1].

#### 2.2.3 XRF

The samples were ground to <75 µm in a Tungsten Carbide milling vessel, roasted at 1000 °C to determine Loss On Ignition (L.O.I) value and after adding 1 g sample to 9 g Li2B4O7 fused into a glass bead. Major element analysis was executed on the fused bead using the ARL9400XP+ spectrometer. Another aliquot of the sample was pressed in a powder briquette for trace element analyses [1]. A blank and certified reference materials were analysed with each batch of samples and the data are provided in Table 3.

#### 2.2.4 ICP-OES

Trace elements data (Table 5) for 21 soil samples were generated using inductively coupled plasma-mass spectrometry (ICP-MS), performed under 1300 W RF power, 15 L/min plasma flow, 2.0 L/min auxiliary flow, 0.8 L/min nebulizer flow, 1.5 L/min for sample uptake rate. A portion of the sample was digested with dilute aqua regia. The digest was then analysed for recoverable Hg by ICP-MS. Based on United States Environmental Protection Agency (USEPA) method 200.2 for the digest and USEPA 200.8 and American Public Health Association (APHA) 3030B for the analysis [9,10]. Electrical conductivity was determined on a slurry of soil in water at a liquid to solid ratio of 2:1. Based on APHA 2510 for the analysis. Inorganic anions (e.g., NO_3_, NO_2_, SO_4_) were determined on a filtered 10:1 water extract of the sample by ion chromatography. The method was based on EPA 300.0 for the extraction and EPA 300.1 and APHA 4110 B for the analysis. 100 ml of water was added to 10 g of soil, shaken for 30 min and allowed to settle for 1 hour. The supernatant was then filtered through a 0.45 µm filter and analysed by discrete analyser. The orthophosphate anion reacted with ammonium molybdate and antimony potassium tartrate (catalyst) under acidic conditions to form a 12-molybdophosphoric acid complex. The complex was then reduced with ascorbic acid to form a blue heteropoly compound. The absorbence of this compound was measured spectrophotometrically at wavelength 880 nm and was related to the phosphate anion concentration by means of a calibration curve. A portion of the sample was digested with dilute aqua regia. The digest as then analysed for recoverable metals, excluding Hg and Si, by inductively coupled plasma optical emission spectrometry (ICP-OES). Based on USEPA method 200.2 for the digest and USEPA 200.7 and APHA 3120 for the analysis [9,10].

#### 2.2.5 Contamination factor

To determine the degree of soil contamination, the trace elements data were compared to the natural background concentrations of the selected elements. This was achieved by calculating a contamination factor (CF) by using Eq. (1), expressed as the ratio of the metal concentration in the sample to the metal concentration in the uncontaminated soil [11].

(1) $$\text{Contamination}\text{Factor}=\left(C_{m}\right)/\left(C_{b}\right)$$

C_m_ is the measured metal concentration in the sample (Table 5), whereas C_b_ represents the background concentration of the metal in uncontaminated soil obtained as the average crustal concentration [12].

#### 2.2.6 Geoaccumulation index

The geoaccumulation index (I_geo_) was calculated using Eq. (2) to determine the level of contamination.

(2) $$\text{Geoaccumulation}\text{Index}=\text{lo}g_{2}\left(C_{n}/1.5B_{n}\right)$$

C_n_ represents the measured metal concentration in the investigated sample, whereas B_n_ provides the background concentration of the metal as determined from the global shale concentration, see the second last row of Table 5 [9]. The value of 1.5 is a constant that accounts for the lithogenic variations in the background concentration for a particular metal in the environment [13,14]. The contamination factor (Eq. (1)), degree of contamination (Table 7), geo-accumulation index (equation 2; Table 8), and soil screening value (Table 10) were critical to determine the soil quality and land use suitability [7,15].

## CRediT Author Statement

**Lowanika Victor Tibane:** Conceptualisation, Methodology, Data curation, Writing – original draft preparation, Visualisation, Investigation. **David Mamba:** Data collection, Analysis, Validation, Reviewing and editing.

## Declaration of Competing Interest

The authors declare that they have no known competing financial interests or personal relationships that could have appeared to influence the work reported in this paper.
